# A life cycle assessment of peritoneal dialysis procurement in Italy: environmental burden and opportunities for improvement

**DOI:** 10.1007/s40620-025-02409-z

**Published:** 2025-09-15

**Authors:** James Larkin, Giulia Ligabue, Gaetano Alfano, Rodrigo Martínez Cadenas, Abass Fehintola, Ingeborg Steinbach, Aycan Yasar, Niccolo Morisi, Marta Arias-Guillen, Marialuisa Caiazzo, Gabriele Donati, Brett Duane

**Affiliations:** 1 School of Dental, Child and Public Health, Trinity College Dublin, Dublin, Ireland; 2 Nephrology Dialysis and Kidney Transplant Unit, Azienda Ospedaliero Universitaria di Modena, Modena, Italy; 3 Universidad Autonoma de Madrid, Madrid, Spain; 4 Centre for Sustainable Healthcare, Oxford, UK; 5 Mozarc Medical, Mirandola, Italy

**Keywords:** Procurement, Peritoneal dialysis, Life cycle assessment, Environmental impact, Plastics, Carbon footprint, Sustainability, Healthcare waste, PVC, LCA

## Abstract

**Background:**

Procurement activities in healthcare, especially within nephrology, contribute significantly to the environmental footprint. In peritoneal dialysis (PD), procurement of consumables such as dialysis bags, tubing, and machines plays a critical role in driving environmental impacts. Previous studies, including those by the National Health Service (NHS), have shown that procurement can account for up to 72% of the healthcare sector’s carbon emissions.

**Methods:**

A life cycle assessment (LCA) was conducted from April to July 2024 at the Nephrology Dialysis and Kidney Transplantation Unit of AOU Policlinico di Modena, Italy, in accordance with ISO 14040/14044 standards. The study focused on procurement-related environmental impacts in automated peritoneal dialysis (APD), based on a standard prescription of two 5L bags overnight and one 2L daytime dwell per day. Products were dismantled to assess materials and modelled using OpenLCA with the Ecoinvent v3.10 database. Transportation, manufacturing, and waste disposal were included within system boundaries.

**Results:**

The 5L Dialysate Bag (used twice daily) had the highest carbon footprint (1515 kg carbon dioxide equivalent [CO_2_-eq/year]), followed by the 2L Bag (457 kg) and Automated Drainage System (286 kg). Primary drivers were long-distance transport, plastic production (especially polyethylene and PVC), and energy-intensive manufacturing. Although the 5L bags are used in greater quantities due to the APD prescription (typically two bags per night), it still showed a lower carbon footprint per litre of dialysate delivered (0.415 kg CO_2_-eq/L) compared to the 2L bag (0.626 kg CO_2_-eq/L). Smaller items like disinfectant sprays and medical kits contributed less individually but were used frequently. Across all categories, plastic production, packaging, electricity use, and incineration were key contributors.

**Conclusion:**

The environmental impact of PD procurement is concentrated in a few high-use, high-impact items. Reduction strategies should target material substitutions, modular product design, and lower-emission transport and energy use. Innovations such as local dialysate mixing, improved waste segregation, and increased recyclability could substantially reduce the environmental burden of PD.

**Graphical abstract:**

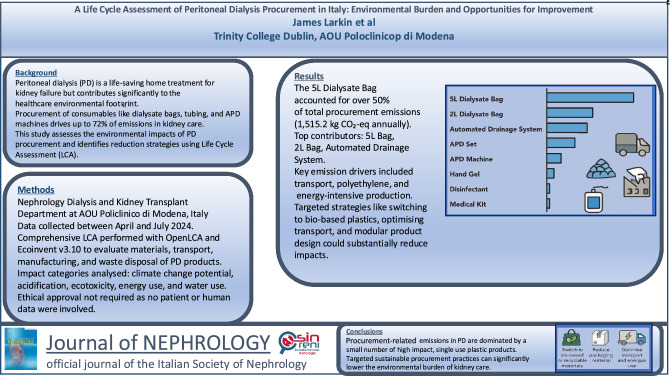

**Supplementary Information:**

The online version contains supplementary material available at 10.1007/s40620-025-02409-z.

## Introduction

Healthcare is increasingly recognised as a significant contributor to climate change, with the procurement of medical devices and supplies playing a central role. Recent studies, including National Health Service (NHS) research, have shown that procurement can account for up to 72% of healthcare's total carbon footprint [1]. In nephrology, where treatments like dialysis are resource intensive, procurement emerges as a critical area for environmental improvement [2].

Kidney failure requires kidney replacement therapies like dialysis or kidney transplantation for survival [3]. Among dialysis options, Peritoneal Dialysis (PD) has emerged as the leading home-based treatment, offering patients more autonomy and convenience [4].

While PD offers many advantages, such as home-based treatment, its environmental impact, particularly from the procurement of materials and medical devices, has become a growing concern. PD requires large quantities of consumables, including dialysis bags, tubing, and connectors, most of which are single use plastics. The environmental burden of manufacturing, transporting, and disposing of these products is substantial [5].

The healthcare sector's contribution to global pollution has led to increasing calls for sustainable practices, especially in procurement, which is a major driver of environmental impact [6]. Life cycle assessment (LCA) offers a comprehensive method for evaluating the environmental footprint of each product and process involved in PD, from raw material extraction to production, distribution, use, and disposal [7]. By assessing procurement in detail, this study aims to identify specific products and processes with the highest environmental impact, enabling the development of targeted strategies to reduce the carbon footprint of PD.

This paper is part of the KitNewCare project, which seeks to reduce the environmental impact of kidney care through sustainable innovations [8].

## Methods

This study was conducted between April and July 2024 at the Nephrology Dialysis and Kidney Transplantation Unit of AOU Policlinico di Modena, Italy, using Life Cycle Assessment based on ISO 14040/14044 [9]. The goal was to evaluate the environmental impacts of procurement activities in automated peritoneal dialysis (APD), covering the full lifecycle of products from raw material extraction to disposal.

### Procurement data collection

A comprehensive inventory of all products used in PD was compiled, with a focus on items with high environmental impacts, such as dialysate bags, tubing, APD machines, and consumables like disinfection sprays and sorbent paper. Data were collected from the AOU Policlinico di Modena to obtain:

- Quantities of each product used annually per patient.
- Supplier and manufacturing details, including production locations and transportation logistics.
- Material composition and associated certifications or product data sheets.

### Material and product analysis

To assess the environmental footprint of individual products:

- Products were disassembled (dismantled into their components, such as plastic bags, connectors, and tubing) to identify materials used.
- Each component was weighed using precision scales, and the material types (e.g., polyethylene, polypropylene, polyvinylchloride (PVC) were identified through safety data sheets and product specifications.
- Packaging materials were included in the analysis to account for their contribution to overall impacts.

### Lifecycle assessment modelling

The environmental impacts of PD products were modelled using OpenLCA software [10]:

- Procurement-related processes, such as raw material extraction, manufacturing, transportation, and disposal, were created for each product.
- The EcoInvent database was used to evaluate environmental impacts across categories such as global warming potential, water use and energy use [11].

### System boundary

**Fig. 1:**
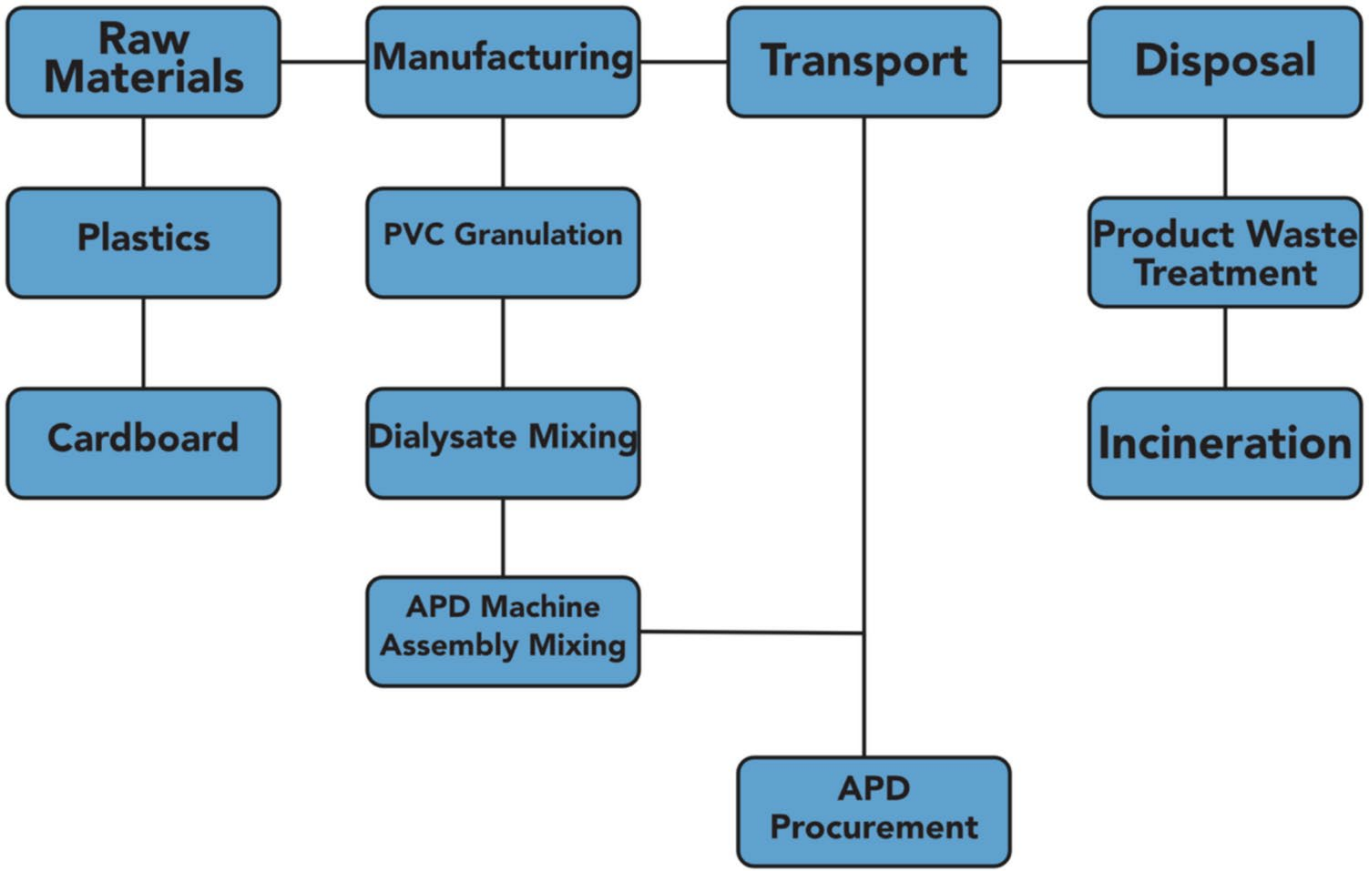
System boundary. *PVC* Polyvinylchloride, *APD* Automated Peritoneal Dialysis

Figure 1 illustrates the system boundaries defined for this study, encompassing all stages from raw material extraction through to end-of-life disposal. This cradle-to-grave approach captures the full environmental burden of each product used in peritoneal dialysis, including specific material types, manufacturing methods, transport distances, usage profiles, and waste treatment.

### Transportation assessment

The transportation stage was assessed to capture the carbon footprint associated with the movement of PD products:

- Shipping distances from manufacturers to the hospital were calculated using online tools such as Google Maps and FluentCargo.com [12, 13].
- The modes of transportation and fuel types were identified through published literature [14].
- Transportation of waste from the hospital to disposal facilities was included in the analysis.

### Disposal pathways

The study analysed waste disposal methods for PD consumables:

- Waste was modelled according to Modena’s hospital protocols. Waste was classified into recyclable and non-recyclable streams based on Modena hospital waste management practices.
- The disposal impacts of incineration, recycling, and landfill were modelled, with data provided by waste management providers and literature sources.
- Home patient waste was collected by third-party contractors and incinerated. Where feasible, we explored potential alternative scenarios (e.g., autoclaving or non-clinical segregation) for discussion [15].

### Stakeholder engagement

Key stakeholders, including clinical staff, admin staff and waste management teams, were asked to verify data accuracy and ensure comprehensive coverage of all procurement-related activities.

### Impact categories

A range of midpoint categories were assessed in OpenLCA. This paper presents the categories with the greatest impact factors: global warming potential, primary energy demand, and water use. Toxicity-related categories were also assessed and are referenced in the Discussion [16]:

- Global warming potential (kg carbon dioxide equivalent [CO_2_-eq]): Total greenhouse gas emissions.
- Primary energy demand (mega-joules [MJ]): Total energy consumed during product lifecycle.
- Water use (m^3^): Water consumed during production and packaging.

### Functional Unit

The functional unit was defined as the complete APD treatment for one patient over one year. The life cycle assessment was based on the most common APD prescription at the hospital: two 5L dialysate bags overnight and one 2L dialysate bag for a daytime dwell, used every day. Equipment quantities and usage patterns were averaged annually per patient based on hospital procurement records. The APD machine’s emissions were annualised based on an assumed 5-year operational lifespan, in line with manufacturer specifications and hospital replacement cycles.

### Ethics

Ethical approval was not required for the project detailed in the document because it involved the environmental assessment of procurement processes and medical devices, rather than research on human participants, patient data, or any other ethical domain requiring institutional review board (IRB) oversight. The focus was on analysing the lifecycle and environmental impacts of products, which does not typically intersect with areas necessitating formal ethical considerations.

## Results

### Product overview

To improve clarity and generalisability, product names have been anonymised into more generic categories. Table 1 describes the updated nomenclature used throughout this section.

**Table 1:** Product descriptions and generic names

Generic name	Description
5L Dialysate Bag	A 5-L dialysate bag with 2.7% glucose concentration used daily in PD. Two bags are taken into account here as per APD prescription
2L Dialysate Bag	A 2-L dialysate bag with 2.7% glucose concentration
Automated Drainage System	PD system component for managing 15L drainage capacity with spike set
Automated PD Set	Cassette-based automated PD set with 8 prong connectors
APD Machine	Automated peritoneal dialysis machine for home use
3L Empty Bag	A 3-L empty drainage bag used in fluid collection
Drainage Set	Tubing and connectors for draining dialysate waste
Hand Washing Gel	Disinfection gel for hand hygiene prior to PD handling
Disinfectant	Surface disinfection spray used before PD exchanges
Medical Kit	Miscellaneous items including surgical masks, gloves, and dressings

**Fig. 2:**
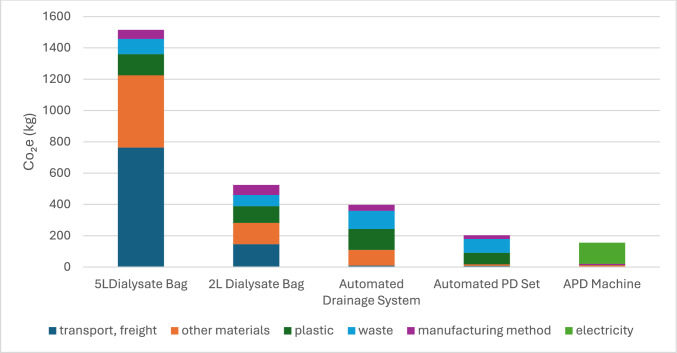
Contribution analysis (carbon emissions)

Total Carbon Footprint (Fig. 2 in Appendix 1).

Figure 2 in Appendix 1 presents the total carbon footprint (kg CO_2_-equivalent) associated with the procurement of PD-related products over one year for one patient.

The 5L Dialysate Bags (two per day as per APD prescription) contributes the largest share (1515.2 kg CO_2_-eq), accounting for over 50% of total emissions. While the 5L dialysate bags had the highest overall primary energy demand and water use, they also showed slightly higher energy and water use per litre when compared to the 2L bags (2.92 MJ/L vs 2.90 MJ/L and 0.0655 m^3^/L vs 0.0479 m^3^/L, respectively). This is taking into account that this is two bags as per APD prescription. This is followed by the 2L Dialysate Bag (457.0 kg CO_2_-eq), and the Automated Drainage System (285.9 kg CO_2_-eq). The Automated PD Set (186.5 kg CO_2_-eq) and APD Machine (151.7 kg CO_2_-eq) also have notable impacts. Smaller but cumulatively important contributors include the Hand Washing Gel (29.9 kg CO_2_-eq), Disinfectant (11.5 kg CO_2_-eq), and Medical Kit (3.3 kg CO_2_-eq).

**Fig. 3:**
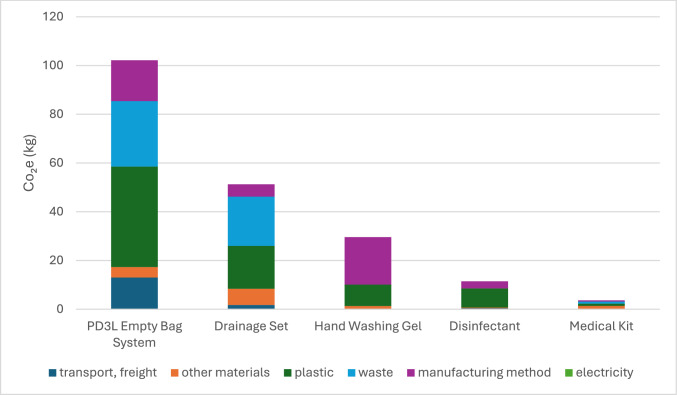
Contribution analysis (carbon emissions)

Carbon emissions contribution analysis (Figs. 2, 3).

In this contribution analysis, “plastic” and “other materials” refer specifically to emissions arising from the production and end-of-life disposal of plastic and non-plastic materials. Plastic-related emissions are highest in the 5L Bag (135.3 kg CO_2_-eq), followed by the Automated Drainage System (133.7 kg) and 2L Bag (107.1 kg). This results from the carbon intensity of producing and incinerating polyethylene and PVC components. The figures presented are ordered by total emissions per product, with each category (transport, plastic, other materials, manufacturing, waste) contributing proportionally to the product’s total footprint. Waste refers to end-of-life emissions, including incineration or landfill. Manufacturing emissions reflect energy-intensive production processes.

Transport dominates for the 5L Dialysate Bags (763.6 kg CO_2_-eq) and 2L Dialysate Bag (145.8 kg CO_2_-eq), due to weight and volume during distribution. Emissions from other materials (e.g., additives, secondary packaging) are particularly high for the 5L Bag (461.4 kg CO_2_-eq). Secondary packaging, particularly corrugated cardboard used for dialysate bag shipments, contributed significantly to emissions. Across one year of treatment, cardboard packaging alone accounted for over 300 kg CO_2_-eq**.** Cardboard is one of the largest contributors to emissions after plastic and transport.

Plastic-related emissions were highest in the 5L Bag (135.3 kg CO_2_-eq), followed by the Automated Drainage System (133.7 kg) and 2L Bag (107.1 kg). This reflects the carbon intensity of producing and incinerating materials such as polyethylene and PVC, rather than product weight alone. Although the 5L bags are used in greater quantities due to the APD prescription (typically two bags per night), it still showed a lower carbon footprint per litre of dialysate delivered (0.415 kg CO_2_-eq/L) compared to the 2L bag (0.626 kg CO_2_-eq/L). This suggests that while the 5L bag has a higher overall impact due to volume and frequency, it remains more efficient per unit of fluid, supporting its continued use under optimised transport and packaging conditions.

Manufacturing contributes substantially across products, with the APD Machine using 134.5 kg of electricity-driven emissions. Waste-related emissions are especially high in the Automated PD Set and Drainage Set, due to incineration of non-recyclable plastics.

Energy resource use (Figs. 5 and 6 in Appendix 1).

Primary energy demand (MJ):

The 5L Dialysate Bags consume 10,648 MJ, followed by 2L Bag (2,119 MJ). High energy demand is associated with both plastic production and long distance transport. Automated Drainage System and Automated PD Set each exceed 2,000 MJ due to multi material complexity.

Water use (Figs. 7 and 8 in Appendix 1).

Water consumption (m^3^): 5L Dialysate Bags lead with 239.2 m^3^ over one year, mostly due to material processing and packaging. Automated Drainage System follows with 26.2 m^3^. Though Medical Kits and Hand Gel have low overall water use, their high water use per kg of product weight makes them inefficient from a water perspective.

## Discussion

This study reinforces the growing body of evidence that procurement activities are a significant driver of environmental impact in healthcare, particularly within dialysis care. As previous research has shown, procurement may account for up to 72% of healthcare’s total carbon footprint [1]. Our life cycle assessment of APD in Italy found that three products alone, the 5L dialysate bag, the 2L dialysate bag, and the automated drainage system, contributed over 75% of the total procurement-related emissions for one patient’s annual treatment. This aligns with prior calls for urgent reform in dialysis-related procurement to enable climate-resilient nephrology care [2].

The 5L dialysate bags were the single largest contributor to emissions, primarily due to high transport emissions, polyethylene use, and energy-intensive manufacturing processes. Importantly, when comparing emissions per litre, the 2L bag is more carbon intensive than the 5L bag (0.626 kg CO_2_-eq/L vs 0.415 kg CO_2_-eq/L), indicating that the use of larger bags may help minimise impact through improved packaging and transportation efficiency when compared to utilising smaller bags of dialysate. However, the contribution from packaging, particularly corrugated cardboard, remained substantial, accounting for over 300 kg CO_2_-eq annually.

Waste management was a key driver of toxicity-related impacts across all products. This was particularly true for PVC-containing products such as tubing and drainage systems, which release dioxins and other harmful compounds during incineration [22]. While some literature suggests PVC is recyclable under the right conditions [17], such systems are not widely implemented in clinical settings. Until they are, transitioning to safer alternatives such as thermoplastic elastomers or silicone, despite their own recycling limitations, may reduce environmental and human health risks [4, 22]. Reclassification of some PD-related waste as non-hazardous, where infection risk is low, could enable increased material recovery and lower the need for incineration [15, 20].

Internationally, alternative clinical waste treatment methods such as autoclaving, microwave disinfection, and mechanical shredding are increasingly adopted to reduce reliance on incineration, which is associated with high energy use [15, 20]. These technologies offer a lower-impact pathway for managing infectious and non-infectious medical waste and could be applied to certain peritoneal dialysis components. For example, the automated drainage system and used PD drain bags are currently treated as clinical waste and incinerated; however, it is unclear whether this classification is always necessary from an infection control perspective. Further research is needed to assess the actual microbiological risk associated with such components. If risks are minimal, reclassifying these items could allow for safer recycling and diversion from incineration, offering substantial environmental benefits.

The use of a broad range of impact categories, global warming potential, energy use, and water consumption allowed us to capture the full environmental burden of procurement activities. While carbon footprint is a dominant concern, a narrow focus on this metric alone would obscure critical trade-offs in energy use and water use [16]. The observed alignment of high-impact products across categories reinforces their environmental impact and highlights the value of full-spectrum life cycle assessments in guiding healthcare sustainability strategies.

In terms of practical reductions, our findings point to several clear opportunities:

- Product redesign: shifting from multi-material to mono-material formats and eliminating PVC where possible. Mono-material formats can improve recyclability by simplifying sorting and reducing contamination.
- Localisation: manufacturing closer to the point of care to reduce transport emissions [18].
- Modular system design: enabling disassembly and more efficient recycling [17].
- Procurement reform: offering dialysate in a wider range of volumes, may reduce unnecessary waste.
- Innovation: exploring on-site dialysate mixing, similar to what has proven effective in haemodialysis, could reduce both transport and packaging impacts [2, 18].
- Transitioning to renewable energy sources for dialysis equipment could further reduce emissions. As our data showed, the electricity required to run an APD machine contributed over 130 kg CO_2_-eq annually. Decarbonising this electricity supply through institutional green energy contracts could substantially lower emissions without changes to the machine design itself.

Our findings align with those reported in a similar study, which assessed the carbon footprint of peritoneal dialysis in China [23]. While their study focused primarily on energy use and patient travel, our work offers a more granular product-level breakdown of emissions. Notably, the overall magnitude of emissions is comparable, but our inclusion of packaging, plastics, and waste management reveals additional hotspots for intervention.

Our results also echo those of another study which examined PD carbon emissions in Australia [17]. Their study emphasised the role of material choices, particularly PVC recycling, in reducing carbon impacts by up to 30%. While such strategies show promise, our study highlights the need for supportive infrastructure to enable recycling and waste reclassification in clinical contexts.

By identifying transport, waste management, and plastic production as the most impactful lifecycle stages, this study offers a roadmap for targeted intervention. These findings support the development of greener procurement strategies and more sustainable nephrology care models, both in Italy and internationally.

This study has several limitations that must be acknowledged. One being that the data collection was limited to a specific timeframe (April to July 2024) and location (Nephrology Dialysis and Kidney Transplantation Unit, AOU Policlinico di Modena, Italy). This does not fully represent the global practices and differences in PD procedures. The Kit New Care project will undertake recreating this study in three other clinical sites. This will eliminate this limitation as comparative studies will be carried out.

The life cycle assessment relied heavily on the accuracy and completeness of the input data. The weights and types of materials used in PD need to be accurate. However, these are subject to measurement and categorisation errors.

Additionally, the environmental impact assessment was constrained by the available impact categories and methodologies in OpenLCA.

## Conclusion

Procurement plays a central role in shaping the environmental footprint of PD. By focusing on the materials, production, and transportation of PD consumables, this paper has identified the main contributors to procurement-related environmental impacts. The dialysis bags, APD machine and tubing products represent the largest sources of emissions and resource use, highlighting the need for focused efforts to reduce their environmental impact.

Sustainable procurement practices, including the adoption of greener materials, optimising production processes, and reducing transportation emissions, are essential to lowering the carbon footprint of PD treatments. As healthcare systems try to become more sustainable, procurement must be a priority area for innovation and improvement in reducing the overall environmental burden of kidney care.

## Supplementary Information

Below is the link to the electronic supplementary material.

Supplementary file1 (DOCX 43 KB)

## Declarations

### Data availability statement

All data generated or analysed during this study are publicly available in the Zenodo repositories: Larkin, J. (2024) ‘Life Cycle Assessment Dataset For Peritoneal Dialysis in Modena’. Brett Duane. 10.5281/zenodo.14258866. Larkin, J. (2024) ‘Product Images for Life Cycle Assessment Dataset For Peritoneal Dialysis in Modena’. Brett Duane. 10.5281/zenodo.14258920.
