# Supplementary material for: A life cycle assessment of peritoneal dialysis procurement in Italy: environmental burden and opportunities for improvement
Source: J Nephrol. 2025 Sep 15;38(8):2311–9. doi: 10.1007/s40620-025-02409-z (PMC12630254; doi:10.1007/s40620-025-02409-z)
Supplement: Supplementary file 1 — Supplementary file1 (DOCX 43 KB) [file 40620_2025_2409_MOESM1_ESM.docx]

# Supplemental Material

Larkin, J. (2024) ‘Life Cycle Assessment Dataset For Peritoneal Dialysis in Modena’. Brett Duane. [doi: 10.5281/zenodo.14258866](https://zenodo.org/records/14258866)

Larkin, J. (2024) ‘Product Images for Life Cycle Assessment Dataset For Peritoneal Dialysis in Modena’. Brett Duane. [doi: 10.5281/zenodo.14258920](https://zenodo.org/records/14258920)

# Appendix 1

Figure 2 Co2 Impact

Figure 5 Contribution Analysis (Energy Resources)

Figure 6 Contribution Analysis (Energy Resources)

Figure 7 Contribution Analysis (Water Use)

Figure 8 Contribution Analysis (Water Use)
